# The Wnt Blows: On the Functional Role of Wnt Signaling in *Mycobacterium tuberculosis* Infection and Beyond

**DOI:** 10.3389/fimmu.2016.00635

**Published:** 2016-12-26

**Authors:** Julius Brandenburg, Norbert Reiling

**Affiliations:** ^1^Microbial Interface Biology, Priority Research Area Infections, Research Center Borstel, Leibniz Center for Medicine and Biosciences, Borstel, Germany

**Keywords:** *Mycobacterium tuberculosis*, Wnt proteins, inflammation, macrophages, toll-like receptors, beta catenin, granuloma

## Abstract

In recent years, it has become apparent that the Wnt signaling pathway, known for its essential functions in embryonic development and tissue homeostasis, exerts immunomodulatory functions during inflammation and infection. Most functional studies indicate that Wnt5a exerts pro-inflammatory functions on its cellular targets, which include various types of immune and non-immune cells. Wnt5a expression has also been linked to the pathogenesis of chronic inflammatory diseases. Activation of beta-catenin-dependent Wnt signaling, e.g., by Wnt3a, has however been shown to limit inflammation by interfering with the nuclear factor kappa-light chain-enhancer of activated B-cells (NF-kappaB) pathway. This review focuses on the regulation of Wnt5a, Wnt3a, and the recently identified Wnt6 and their functional role in bacterial infections with a primary focus on pulmonary tuberculosis, a leading infectious cause of morbidity and mortality worldwide.

## Tuberculosis (TB): Overview and Pathogenesis

With 1.5 million fatalities in 2014, TB ranks alongside with the human-immunodeficiency virus (HIV/AIDS) as the leading-cause of death from infectious disease worldwide (1). The causative agent of TB belongs to the genus *Mycobacteria* and to the Mycobacterium tuberculosis complex, which comprises of several closely related pathogenic sub-species (2). *Mycobacterium tuberculosis* (*M. tuberculosis*) is a non-motile, slow growing, gram-positive bacterium with a lipid-rich cell wall. The pathogen is adapted to an intracellular lifestyle, allowing it to survive and replicate within its main host cell, the macrophage (3, 4). TB primarily affects the lung (pulmonary TB) and is usually transmitted by inhalation of very small bacteria-containing droplets (aerosols).

Having entered the lung, *M. tuberculosis* is ingested by alveolar macrophages and elicits a localized inflammatory response, which, by production of a range of cytokines and chemokines, leads to early recruitment of mono- and polymorph nuclear cells from neighboring blood vessels to the infectious foci (5, 6). This leads to the formation of a cluster of immune cells termed granuloma, the hallmark of TB infection. Initial stages are mainly characterized by an amorphous mass of tissue resident- and monocyte-derived macrophages (7). Naïve T-cell differentiation is driven by antigen-presenting dendritic cells that have migrated to the local lung-draining lymph node (8). Subsequently, antigen-specific T-cells enter the site of infection and the granuloma lesion stratifies. During progression of disease, some cells differentiate into multinucleated giant cells and foamy macrophages. Infected macrophages in the core of the granuloma are enclosed by uninfected and foamy macrophages, which, in turn, are surrounded by varying fractions of T- and B-lymphocytes in the periphery (9–11). In “late” phase granulomas, a fibrous cuff may separate macrophages from lymphocytes, while the center of the granuloma necrotizes, generating the liquefied core termed “caseum.” A functional disintegration of the granuloma ultimately leads to the release of viable, extracellular mycobacteria into the airways. This facilitates transmission of disease by coughing and sneezing of the patient.

## TB: Ligands, Receptors, and Signal Transduction Pathways

Cell wall components such as lipoproteins, lipoarabinomannans, and mycolic acids represent conserved mycobacterial structures, which are recognized by pattern recognition receptors (PRRs) of the immune system [recently extensively reviewed in Ref. (12)]. Among these, the family of Toll-like receptors (TLRs) significantly contributes to the initiation of the first line defense response against the invading pathogen. Engagement of TLRs initiates downstream signaling events, which are orchestrated by various adaptor proteins, including the myeloid differentiation primary response gene 88 (MyD88) (13, 14). TLR-MyD88-signaling leads to the translocation of nuclear factor kappa-light chain-enhancer of activated B-cells (NF-kappaB) into the nucleus, which induces the transcription of a variety of cytokines and chemokines, including interleukin (IL)-12 and tumor necrosis factor alpha (TNF-alpha). TNF-alpha is a pro-inflammatory cytokine mainly secreted by macrophages, which was shown to mediate early leukocyte recruitment and granuloma formation in mice (15–18). TNF-alpha expression is critically linked to host protection, since latently *M. tuberculosis*-infected individuals (19) that received anti-TNF reagents to treat inflammatory diseases (e.g., rheumatoid arthritis) showed an enhanced TB reactivation risk (20). TLR-induced macrophage activation and the release of cytokines and chemokines contribute to the initiation of adaptive immunity (21). Antigen-specific T-helper cells type 1 (Th1), migrating to the site of infection, secrete interferon-gamma (IFN-gamma), which is critical for the host defense against *M. tuberculosis* (22, 23). It induces a profound transcriptional remodeling of macrophages (24), which sensitizes cells for a more rapid and heightened response to TLR-ligands (25). Synergistic action of IFN-gamma and TLR-ligands “fully” activates macrophages (also referred to as classical activation of macrophages) (25) and enables cells to exert tuberculostatic functions (26, 27). Although it has been shown that TLR2 and Myd88 are not essentially required for the induction of adaptive T cell responses, MyD88 - but not TLR2, TLR4, and TLR9 - is critical for triggering macrophage effector mechanisms central to anti-mycobacterial defense (28, 29).

## Wnt Signaling: Overview

The Wingless/Integrase 1 (Wnt) signaling pathway, which is evolutionary highly conserved in multicellular eukaryotic animals (metazoa), regulates basic cellular processes such as proliferation apoptosis, differentiation, polarization, and motility [reviewed in Ref. (30–32)]. Exerting fundamental functions on cells, Wnt signaling plays a crucial role during embryogenesis, but also throughout adult life by maintaining homeostasis in virtually every tissue and organ. Regarding the lung, as the organ primarily affected during TB, Wnt signaling regulates various processes during organogenesis including branching morphogenesis and regional specialization of the epithelium and mesenchyme thereby shaping the architecture of the airways not only in mice but also in other animal species [reviewed in Ref. (33, 34)]. Moreover, dysregulation of Wnt signaling has been linked to lung diseases such as lung cancer, pulmonary fibrosis, or pulmonary arterial hypertension (33, 34).

With regard to the immune system, there is evidence that Wnt signaling is necessary for normal hematopoiesis (35). Moreover, the work of the last years shows that Wnt signaling affects immune cell function during inflammation and infection, demonstrating that this ancient and conserved signaling pathway also regulates processes beyond development and homeostasis. Recent work indicates that Wnt ligands and downstream signaling molecules shape inflammation and the host response to pathogens by modulating various immune cell functions, including cytokine production, immune cell migration, or differentiation [reviewed in Ref. (36–38)]. The heterogeneity and complexity of Wnt regulated functions are reflected by the presence of numerous receptors, which integrate the signals of various ligands, altogether determining cellular functions of target cells.

## Wnt Signaling: Ligands, Receptors, and Signal Transduction Pathways

The human and the mouse genome harbor 19 independent Wnt genes (39), which show distinct expression patterns depending on the cellular context. Wnt genes encode for Wnt ligands which are cysteine-rich, glycosylated, lipid-modified, and secreted proteins with a molecular weight of approximately 40 kDa (40). Wnts can act as short-range mediators as they stick to secreting cells or the extracellular matrix (41). To exert their action across a distance in tissues (e.g., as morphogen), it was proposed that Wnts are associated with lipoprotein particles (42). It has also been demonstrated that they are released in small extracellular micelles from endocytic origin, the exosomes (43). This extracellular vesicular transport enables Wnts to bypass long distances and, as they are exposed on the surface of the exosome, to efficiently bind to their receptors and induce signaling in their target cells. Wnt proteins mainly engage Frizzled (Fzd) family members to transduce signals into target cells. In addition, several coreceptors such as lipoprotein-receptor-related protein (LRP) participate in Wnt signaling (44). The 10 mammalian Fzd proteins are seven-transmembrane receptors, belonging to the family of G-protein-coupled receptors (45–47). Fzd receptors comprise of three different domains, the large N-terminal cysteine-rich extracellular region (CRD), the central core with seven hydrophobic membrane spanning alpha helices, and the cytoplasmatic region. Recently, the successful crystallization of Xenopus Wnt8 complexed to the CRD of Fzd8 gave first insights into the structural basis of ligand-receptor binding (48). For signal transduction inside the target cell, the cytoplasmatic region of Fzd receptors mediates binding of adaptor proteins such as dishevelled (Dvl) or heterotrimeric G-proteins (49, 50). To date, at least three distinct signaling cascades are known to be induced by ligand-receptor binding: the Wnt/beta-catenin pathway, the beta-catenin independent Wnt/Ca^2+^ pathway, and Wnt/Planar cell polarity pathway [reviewed in Ref. (51) and described below].

## Wnt Signaling Promoting Inflammation - Wnt5a

Our group was the first who linked the activity of Wnt signaling to infectious disease mechanisms operative in pulmonary TB. We showed that Wnt5a and its putative receptor Frizzled5 (Fzd5) are present in lung biopsies of TB-patients, that mycobacteria induce Wnt5a in macrophages in a TLR-NF-kappaB dependent manner, and that Wnt5a exerts a distinct immunomodulatory function on immune cells (52). This observation was well in line with earlier findings by Sen et al., who had observed an enhanced presence of Wnt5a in inflamed synovial tissue of rheumatoid arthritis patients and had assigned a functional role for Wnt5a in the pathogenesis of this chronic inflammatory disease (53). During the last 10 years, an increased Wnt5a expression was observed in a variety of inflammatory disease settings such as skin lesions of patients with psoriasis vulgaris (54) or cutaneous lichen planus (55), obesity-induced adipose tissue inflammation (56, 57), atherosclerotic lesions (58), and in the sera of patients with septic shock (59). With regard to infections, Wnt5a induction has been described in response to a variety of pathogens (60, 61). While mostly human macrophages were studied, Bansal et al. could show that Wnt5 is expressed in murine macrophages after infection with *Mycobacterium bovis* BCG - but not with *Mycobacterium smegmatis* (62). Notably, the authors show that the induction of Wnt5a is also dependent on the presence of TLRs. Also engagement of the C-type lectin dectin-1 was shown to drive Wnt5a formation in macrophages (63). In addition to human and murine cells, an increased expression of Wnt5a was also observed in *Mycobacterium marinum*-infected adult zebrafish (64), demonstrating that induction of Wnt5a upon infection is a conserved mechanism.

Beside the abovementioned TLRs and dectin-1, a whole variety of PRRs contribute to host cell activation by pathogenic mycobacteria and were shown to be involved in mounting an anti-mycobacterial response. This includes the macrophage inducible C-type lectin (MINCLE), macrophage scavenger receptor A (SRA), macrophage receptor with collagenous structure (MARCO), and the intracellularly located nucleotide-binding oligomerization domain-containing protein 2 (NOD2), NLR family, pyrin domain-containing 3 (NLRP3), as well as the dsDNA sensor cyclic GMP-AMP synthase (12). It is currently not known whether ligand binding to these receptors and the resulting downstream signaling pathways contribute to the *M. tuberculosis*-induced expression of Wnt proteins in macrophages.

The work of many independent laboratories has shown that Wnt5a mainly exerts pro-inflammatory functions on its cellular targets, which include non-immune cells (65, 66) as well as various types of immune cells (52, 56, 59, 67–69). In macrophages, Wnt5a has been shown to activate the Wnt/Ca^2+^ pathway (59), which leads to an increase in cytoplasmic Ca^2+^ levels thereby activating Ca^2+^-dependent factors such as Proteinkinase C, Ca^2+^/calmodulin-dependent kinase II (CaMKII), and Calcineurin. Independent laboratories have shown that there is an intense crosstalk between the Wnt5a-induced signaling cascade and the NF-kappaB as well as certain mitogen-activated protein kinase pathways (56, 70, 71). Under homeostatic conditions, the Wnt5a–Fzd5–NF-kappaB (p65) signaling axis was shown to sustain macrophage immune functions (70). It was demonstrated that Wnt5a stimulates phagocytosis, but does not affect bacterial killing by macrophages (68). Wnt5a induces endothelial inflammation (65) and contributes to CXC chemokine 12-ligand mediated T-cell migration (67). In the context of mycobacterial infections, Wnt5a exerts important functions by bridging innate and adaptive immunity: the antigen response of peripheral blood mononuclear cells (PBMCs) of *purified protein derivative of M. tuberculosis* (PPD) reactive healthy donors is altered by blocking Wnt5a or its putative receptor Fzd5 (52). Both, the production of the cytokine IL-12, critical for dendritic cell migration and T cell priming during TB infection (72), and T cell derived IFN-gamma, critical for “full” activation macrophages, are significantly reduced in PBMCs incubated either with a neutralizing antibody or an antiserum against Wnt5a or Fzd5, respectively (52). These findings suggest that Wnt5a plays an important regulatory role during the host response to *M. tuberculosis* infection by activating innate immune cells, which in turn directly affects adaptive immune cell function. However, Wnt5a expression and its pro-inflammatory activity have also been linked to the pathogenesis of multiple chronic inflammatory diseases, including rheumatoid arthritis (66), psoriasis (73), colitis (69), atherosclerosis (74–76), and obesity (56, 57). With regard to TB, however, it remains elusive whether excessive Wnt5a formation may contribute to the *M. tuberculosis*-induced immunopathology.

## Wnt Signaling Limiting Inflammation - Wnt/Beta-Catenin Signaling

Wnt/beta-catenin signaling is characterized by Wnt ligand binding to Fzd and LRP coreceptors, leading to the recruitment of heterotrimeric G-proteins and the adaptor protein Dvl to the membrane (77). Subsequently, the so-called “destruction complex,” which continuously tags beta-catenin for proteasomal degradation by the activity of kinases such as glycogen-synthase kinase-beta (GSK3beta), dissociates (78). The accumulation of beta-catenin in the cytoplasm and its translocation into the nucleus leads to activation of gene expression mediated by the transcription factors T-cell factor and lymphoid enhancer-binding factor (TCF/LEF).

In *M. tuberculosis* aerosol infection experiments, we systematically screened the mRNA expression of all 19 *Wnt* genes (79) and Fzd receptors (80) in lungs of infected mice. We were able to detect all 19 *Wnt* genes at all time points analyzed but found a very prominent expression pattern. The mRNA expression of *Wnt2, 2a, 3a, 4, 5a, 7a, 8a*, and *10b* was significantly reduced during infection whereas Wnts *5b, 8b, 9a, 9b, 11*, and *Wnt16* were not regulated. These results complement data from an earlier study, which demonstrated that also the mRNA expression of the majority of Fzd receptors is downregulated after *M. tuberculosis* infection (80). The bacterial burden in the lung is characterized by a rapid increase of bacterial numbers between day 1 and day 21 postinfection (p.i.) followed by a plateau of *M. tuberculosis* replication until day 42. During the first 3 weeks, a massive inflammatory response in the lung in response to infection is established in order to limit the growth of the pathogen. This is characterized by the induction of a robust T cell response and the formation and release of pro- and anti-inflammatory mediators. Thus, when analyzing gene expression in the lung, we observe an inverse correlation of Wnt expression and the formation of pro-inflammatory factors. Although the individual signaling induced by a given Wnt protein needs to be studied on the cellular level, most of these downregulated Wnt factors were previously associated with Wnt/beta-catenin signaling. This is well reflected by a reduced expression of the known Wnt/beta-catenin target gene and feedback regulator Axin2 (81), which was reduced by approximately 50% at days 21 and 42 p.i. (80). In line with our data, in the lung of *Streptococcus pneumoniae*-infected mice, a reduced Wnt activity has been observed by kinase activity profiling (82).

Notably, *M. tuberculosis*-infected mouse lungs *in vivo* and -infected macrophages *in vitro* showed significantly enhanced expression levels of the receptor Frizzled1 (Fzd1) (80). Moreover, Fzd1 surface expression was increased by stimulation of macrophages with the key cytokine IFN-gamma. Initially, we were puzzled by this observation, because Fzd1 has been clearly linked to Wnt/beta-catenin signaling, while IFNgamma is known to boost pro-inflammatory responses of macrophages to TLR-ligands (25). In a variety of *in vivo* studies, it was shown that mimicking activation of Wnt/beta-catenin signaling by administration of a GSK3beta inhibitor potently suppresses pro-inflammatory responses and thereby protects animals from pathophysiological conditions (83–86). Due to the fact that epithelial cells in the lung did heavily stain for Wnt3a (80), we hypothesized that upregulation of Fzd1 renders the macrophages more sensitive to Wnt/beta-catenin signaling. And indeed we observed that macrophages reacted to Wnt3a stimulation or GSK3beta inhibition with beta-catenin stabilization and prominently enhanced Axin2 transcript levels, which was accompanied by a significant reduction of *M. tuberculosis*-induced TNF-alpha formation. This demonstrated that the Wnt3a-induced signal interferes with the *M. tuberculosis*-induced TLR2-dependent macrophage activation *in vitro*. To our knowledge, this was the first observation of a counter-regulation of TLR/NF-kappaB/IFNgamma and Wnt/beta-catenin signaling in infected macrophages. Interestingly, this phenotype of a Wnt/beta-catenin negative feedback loop, which represses TLR-triggered inflammatory responses, was also observed in alveolar epithelial cells (87). Along the same line, another beta-catenin-dependent Wnt, Wnt2 has been shown to inhibit enteric bacterial-induced inflammation also in intestinal epithelial cells (88).

Depending on the cellular context, other Wnt-independent pathways may also lead to inhibition of GSK3beta, as e.g., shown for the integrin-linked kinase, an enzyme regulated by integrin signaling, which was shown to stabilize beta-catenin and to activate its target genes (89). It also needs to be considered that infections with pathogenic microorganisms (including *M. tuberculosis*) are often associated with severe tissue damage. This leads to the release of various intracellular factors such as adenosin triphosphate (ATP), heat shock proteins, mitochondrial components and several alarmins, a group of proteins which include high-mobility group box 1 protein, IL-1α, IL-33, and the Ca^2+^-binding S100 proteins [reviewed in Ref. (90)]. Recent studies suggest that some of these factors indeed interfere with GSK3beta activity and affect beta-catenin stabilization and the expression of related target genes. Guo et al. observed that activation of P2X7R by BzATP caused the death of alveolar epithelial cells type I by suppressing Wnt/beta-catenin signaling through stimulating GSK3beta (91). In contrast, S100 A8/A9 proteins have been shown to induce beta-catenin signaling in macrophages (92).

## Opposing Roles of Wnt5a and Wnt/Beta-Catenin Signaling in Inflammation

In a model of endotoxic shock, lung homogenates from rats that received lipopolysaccharide (LPS) exhibited a decrease in levels of Wnt3a, Fzd1, phosphorylated GSK3β at Ser9, total β-catenin, and nuclear beta-catenin, demonstrating that major constituents of the Wnt3a signaling machinery in the lung were downregulated. In contrast, Wnt5a, Fzd5, total CaMKII, and phosphorylated CaMKII were upregulated, indicating that Wnt5a dependent, pro-inflammatory Ca^2+^/calmodulin signaling is strongly activated in endotoxemic rats (93). These findings are corroborated by Villar et al. who observed that lungs of septic animals and humans are characterized by acute lung inflammation, collagen deposition, and marked increase of Wnt5a (94). These data show that in certain acute inflammatory situations pro-inflammatory Wnts, such as Wnt5a are induced, whereas Wnt/beta-catenin associated signals are downregulated. As mentioned above mimicking activation of Wnt/beta-catenin signaling by administration of a GSK3beta inhibitor protects animals from pathophysiological conditions. This was shown in mice receiving the TLR4 agonist LPS which were protected from endotoxin shock (84, 86), and in Francisella-infected mice, which were protected against tularemia (95). On a cellular level there is very likely a tightly controlled signal integration of both pathways. Not only in primary macrophages (37, 80), but also in dendritic cells beta-catenin has been shown to regulate the balance between inflammatory and regulatory responses. Beta-catenin in intestinal dendritic cells was required for the expression of anti-inflammatory mediators and the stimulation of regulatory T cell induction, while suppressing inflammatory effector T cells (96). Furthermore, ablation of beta-catenin expression in dendritic cells enhanced inflammatory responses and disease in a mouse model of inflammatory bowel disease. Thus, beta-catenin signaling programs dendritic cells to a tolerogenic state, regulating, and limiting the inflammatory response (96). During the process of infection, this balance is very likely to change. Once the acute phase of an infection has passed, it is likely that Wnt/beta-catenin signals regain more and more activity over time as it is known that its downstream effects are also associated with termination of infection or inflammation and the reestablishment of homeostasis. The latter has been shown in bacteria-induced injury of the urogenital tract (97) during which activation of Wnt/beta-catenin signaling promotes the regenerative response to bacterial injury. This is well in line with the observation that Wnt signaling induces epithelial differentiation during cutaneous wound healing (98).

## Wnt6 and Its Role During Disease

In the murine lung, only 3 out of 19 Wnt ligands were upregulated after *M. tuberculosis* infection (79). Whereas *Wnt1* and *Wnt10a* were expressed to a significantly higher extent only at day 42 p.i., mRNA expression of *Wnt6* was increased significantly at days 21 and 42 p.i. (79). Performing immunohistochemical analyses of lung tissue sections, we found Wnt6 in the *M. tuberculosis*-infected lung at both time points analyzed, but not in the uninfected lung. To our knowledge, this was the first description of Wnt6 expression in the lung. Notably, Wnt6 expression was exclusively found in *M. tuberculosis*-infected cells within granulomatous lesions, which could be stained for CD68, a well known marker for myeloid cells such as monocytes and macrophages. Subsequent *in vitro* studies in primary cells showed that Wnt6 is macrophage-derived immunomodulatory factor, which impairs expression and release of the key cytokine TNF-alpha during mycobacterial infections (79). Remarkably, Wnt6-conditioned medium (Wnt6 CM) added to primary murine macrophages induced c-Myc expression, while c-Myc expression was significantly reduced in *Wnt6*-deficient cells when compared to respective control cells. The transcription factor c-Myc is a global regulator of chromatin which regulates diverse cellular processes including cell cycle (99). Indeed, Wnt6 CM also enhanced Ki-67 expression levels and ^3^[H]-Thymidin incorporation into cells, indicating that exogenous Wnt6 exerts proliferative effects on primary murine macrophages. However, exogenous Wnt6 did not enhance Axin2 mRNA levels or induce beta-catenin accumulation. These findings were surprising because (i) c-Myc is described as a well-known Wnt/beta-catenin-dependent target gene (100) and (ii) many reports describe that Wnt6-induced signaling is beta-catenin dependent (101, 102). Recent insights into the structural basis of ligand-receptor interaction (48), showing conservation of interacting binding structures, suggest that the induced signaling and function of a certain Wnt ligand might not be predicted by its structure *per se*. It is known that Wnt5a can initiate discrete signaling pathways through the activation of distinct receptors (103). Moreover, when studying binding affinities of purified Wnt ligands to different Fzd CRDs, Wnt3a, -4, and -5a are functional binding partners for Fzd2, 4, and 5 with appreciable affinity (104). Only Wnt3a has the same effect on downstream signaling with each of the three receptors (104, 105), while it is described that the Wnt ligands, Wnt4 and Wnt5a, are also able to induce beta-catenin-dependent signaling in fusion with specific Fzds and coreceptors (105). These findings indicate that, although Wnt ligands may have a putative bias for individual signaling pathways (104), downstream consequences depend on the presence of receptors, coreceptors, competing ligands, and signaling components in the target cell and a given tissue. The receptor and coreceptor context in which Wnt6 engages a particular signaling pathway in macrophages is, however, not known to date.

So far, Wnt6 was described in the context of early embryogenesis (106, 107), during mouse embryonic gut (108), stomach (109), kidney (110), and heart (111) organogenesis as well as in tooth morphogenesis (112). Dysregulation of Wnt6 has been shown to be related to carcinogenesis: single nucleotide polymorphisms (SNPs) in the Wnt6 gene could be associated with enhanced risk for developing colorectal adenomas (113). Wnt6 was also described to contribute to chemoresistance of gastric cancer cells (114). Both findings are in line with the observed proliferative effect of Wnt6 on macrophages.

The fact that Wnt6 is also induced by conserved microbial structures suggests that it may play a role not only during mycobacterial infections but also in the context of other infectious diseases. Indeed, Liu et al. reported that *Salmonella* infection induces Wnt6 expression in intestinal epithelial cells (115). In a non-infectious context, Choy et al. showed that Wnt6 expression positively correlates with the Th2 signature observed in mild-to-moderate asthmatics (116). Collectively, the currently available data demonstrate that Wnt6 is expressed during *M. tuberculosis* infection, but may also be expressed in the context of other inflammatory and infectious disease settings. With regard to function, our study identifies Wnt6 as a macrophage-derived anti-inflammatory and pro-proliferative factor, which is likely to influence immune cell function during TB infection (79).

## How Much Wnt is in the *M. Tuberculosis* Granuloma?

**Figure 1:**
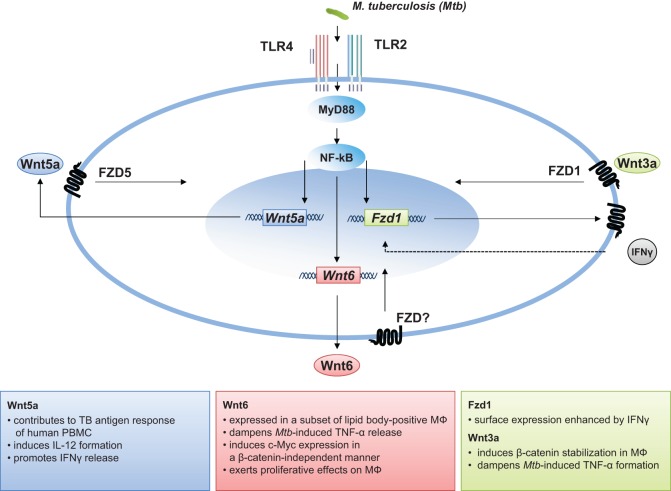
**The role of Wnt signaling in *M. tuberculosis* infection**.

The granuloma is the key pathologic feature of TB. Granulomas serve as an immunological microenvironment, which enable the host to contain microbes and control disease and, at the same time, act as a niche for the pathogen in which the bacteria can persist or even grow (117, 118). As a highly dynamic structure, the granuloma changes appearance during the course of disease. In addition, there is substantial granuloma heterogeneity, as granulomas with varying cellular composition exist within the same animal or patient (119, 120). It is widely accepted that the activation of robust Th1-dominated adaptive immune responses characterized by (i) efficient migration of antigen-specific T-cells into the lung, (ii) a profound IFNgamma release, and (iii) high numbers of classical activated macrophages at the site of infection are essential to efficiently restrict bacterial growth (22, 23, 121, 122). However, uncontrolled pro-inflammatory Th1 responses causing excessive tissue damage, which affects the functional integrity of the granuloma, may be detrimental for the host (123). Available data demonstrate that Wnt signaling is regulated during lung inflammation and suggest that the activity of beta-catenin-dependent Wnt signaling affects the inflammatory balance during inflammation and infection. Studies in non-human primates showing that even during active disease some lesions are sterile (119, 120), and recent results from patients showing that pro- and anti-inflammatory signals are physically segregated within each granuloma (124), suggest that local mechanisms rather than systemic inflammatory responses dictate the functional integrity of the granuloma. Moreover, it seems likely that other factors than cytokines present within the segregated microenvironments of the granuloma contribute to its “homeostasis.” Wnts act on immune cells in different ways as described above. This does not only include immunomodulatory functions as shown for Wnt5a and Wnt3a, but affects immune cell differentiation and proliferation as shown for Wnt6 (summarized in Figure 1). Although *in vivo* studies are missing, current data on Wnt6 imply that this factor, acting as an anti-inflammatory and pro-proliferative mediator *in vitro*, attenuates tissue inflammation and drives macrophage differentiation during TB infection. In addition Wnt6 is expressed a subset of lipid body-positive macrophages in *M. tuberculosis*-infected. (79). Foamy macrophages are considered as key cells, which constitute a nutrient-rich reservoir for *M. tuberculosis* persistence and contribute to tissue pathology (125, 126). There is increasing evidence that Wnt signaling is of critical importance for cellular lipid metabolism (127). Mani et al. identified Wnt signaling as a regulator of plasma lipid composition and as target for treatment of hyperlipidemia (128). Whether there is a link between Wnt6 and the formation of foamy macrophages in *M. tuberculosis* infection remains to be shown. The impact of Wnts may even target other important pathways in the host mycobacteria interaction. Activation by Wnt3a augments *M. bovis* BCG-induced apoptosis in RAW264.7 macrophages (129), indicating a modulation of cellular cell death pathways by Wnt/beta-catenin signaling in macrophages. In addition, the characterization of the microRNA profile circulating of patients with active pulmonary TB has led to the identification of increased levels of miR-29a (11.9- and 5.2-fold) in the TB serum and in the TB sputum, when compared to the expression of the controls (130). This is an important observation since MiR-29a directly targets negative regulators of Wnt signaling (131). Taken together, although Wnt expression is detected within tuberculous lesions, it is currently not clear how much of a given Wnt is “seen” by a macrophage and other immune cells in the granuloma and how this impacts the outcome of disease.

## Pathogens Exploiting Wnt Signaling

Whereas Wnt/beta-catenin signaling is host protective in many of the above mentioned infection models, it has also been shown that certain pathogens harness Wnt signaling components to promote infection. In a very recent study, *Ehrlichia chaffeensis*, an obligate intracellular bacterium responsible for the emerging life-threatening zoonosis human monocytotropic ehrlichiosis exploits beta-catenin-dependent and beta-catenin-independent host Wnt signaling pathways to stimulate phagocytosis and promote intracellular survival (132). Ehrlichial tandem repeat proteins were identified to be responsible for these effects. siRNA knockdown of several Wnt signaling pathway components or targets influences significantly reduced ehrlichial infection in human THP1 macrophages. A second example is *Chlamydia trachomatis* (*Ctr*) representing another gram-negative bacterium which exploits Wnt/beta-catenin signaling during infection. These bacteria disturb epithelial tissue homeostasis in fallopian tubes. The authors demonstrate that acute *Ctr* infection activates the paracrine Wnt signaling pathway, leading to profound disruption of epithelial cell structure and function, which facilitates the dissemination of damage beyond that of infected cells (133).

Also viruses have been shown to directly interfere with the Wnt signaling cascade. The activation of Wnt signaling has been demonstrated for the human polyoma virus JC (134), Epstein–Barr virus (135), and Kaposi’s sarcoma-associated herpesvirus (136), being just a few examples of viruses which are known to lead to tumor development, an issue very recently reviewed (137). With regard to viral infections of the airways, adenoviruses are known to strongly interfere with the Wnt signaling machinery as several factors are heavily regulated during infection (138). In a very recent study, the 1918 influenza virus PB2 protein was shown to be a virulence factor interfering with inflammatory and Wnt-mediated signaling in mice. Both, beta-catenin-dependent and -independent Wnt signaling pathways were repressed and associated with impaired lung regeneration and repair (139). Taken together, this illustrates that depending on the infection mechanism and the infected host cell, pathogens may exploit the Wnt signaling machinery in a very specific manner.

## Wnts and the Susceptibility to Infectious Disease

With regard to infectious diseases, SNPs in the Wnt signaling cascade have been mainly associated with the risk of cancer development upon infection. It was shown that beta-catenin mutations are frequent in human hepatocellular carcinomas associated with hepatitis C virus (HCV) infection (140). In addition, WNT signaling pathway gene polymorphisms are related to the risk of hepatic fibrosis and inflammation in HCV-infected patients (141). In *Helicobacter pylori* infection, SNPs of *MMP-9* -1562/*TIMP-1* 372 have been associated with an enhanced risk of gastric intestinal metaplasia (142). With regard to TB there are indications that certain SNPs are associated - not just with the consequences of the disease as described above but with disease susceptibility (143). Based on studies in two cohorts, the authors identified three SNPs in the *CTNNB1* gene resulting in lower beta-catenin mRNA and protein levels, which were associated with a decreased risk of developing TB. By contrast, two SNPs associated with SFRP1, which were associated with higher C-reactive protein levels that were significantly associated with TB risk in a Chinese Han population, but not in a Tibetan group. These data are the first that suggest that Wnt pathway polymorphisms may influence TB susceptibility and host immune response. It will be interesting to see whether these observations will be seen in other, larger cohorts of different ethnicity, or whether this is a characteristic feature of these specific ethnic groups studied. To date, this recent study is to our knowledge the only one which directly links SNPs in Wnt signaling related genes to the susceptibility towards an infectious disease.

## Concluding Remarks

Pulmonary TB is one of the most complex infectious diseases known, characterized by severe chronic inflammation being important for bacterial growth restriction but also being responsible for severe immunopathology. In the last 10 years, it has become clear that the immune function during *M. tuberculosis* infection is regulated by members of the Wnt signaling cascade. And still new factors and related functions are being identified. A simplified view on the systemic expression and functional role of each of the 19 individual Wnts in *M. tuberculosis* infection is currently not possible, since to our understanding local determinants define either the induction or repression of a given Wnt signaling factor and its related functions. The detailed characterization of the underlying signaling pathways and the identification of molecular interactions will lead to better understanding of the disease mechanisms and may even lead to the identification of new target structures in order to develop a shorter and more efficient therapy of pulmonary TB.

## Author Contributions

All the authors listed have made substantial, direct, and intellectual contribution to the work and approved it for publication.

## Conflict of Interest Statement

The authors declare that the research was conducted in the absence of any commercial or financial relationships that could be construed as a potential conflict of interest.
